# Pharmaceutical Expenditure and Burden of Non-communicable Diseases in Serbia

**DOI:** 10.3389/fphar.2016.00373

**Published:** 2016-10-13

**Authors:** Aleksandra Kovacevic, Nemanja Rancic, Zoran Segrt, Viktorija Dragojevic-Simic

**Affiliations:** ^1^Centre for Clinical Pharmacology, Military Medical Academy Medical Faculty, University of DefenseBelgrade, Serbia; ^2^Military Medical Academy Medical Faculty, Management of the Military Medical Academy, University of DefenseBelgrade, Serbia

**Keywords:** non-communicable diseases, pharmaceutical expenditure, Serbia, gross domestic product, drug utilization

In the low- and middle-income countries some non-communicable diseases (NCDs), such are cardiac diseases, strokes, chronic lung diseases, certain cancers, and diabetes, tend to overtake the morbidity and mortality of poverty diseases (Adams and Butterly, 2015; Jakovljevic and Getzen, 2016). Such diseases, conditionally labeled as diseases of affluence (Nutrition Health Topics, 2016), are growing rapidly in the developing countries, making almost 80% of deaths due to NCDs (New WHO report: deaths from noncommunicable diseases on the rise, with developing world hit hardest, 2011). Until a century ago, infectious diseases were the leading cause of morbidity and mortality worldwide. For example, in the United States at the beginning of the twentieth century, the crude death rate for the infectious diseases amounted to 800 per 100,000 population per year, reaching nearly 1000 during influenza pandemic at the end of the WWII. Afterwards, significant drop in the morbidity and mortality due to infectious causes was noticed, mostly as a consequence of a variety of the conducted preventive measures in sanitation and hygiene area, vaccination strategies and the development of different antimicrobial agents (Achievements in Public Health, 1900–1999: Control of Infectious Diseases, 1999; Wang et al., 2016). Due to the globalization, the flow rate of people, ideas and financial capital increased worldwide (Jakovljevic et al., 2016).

Non-communicable diseases have achieved high morbidity and mortality rates, first in the developed (affluent) and afterwards in the low- and middle-income countries, dislodging the rich-countries exclusivity premise. On the contrary, the wealthier people live, socially and economically, the better their health (Fair Society Healthy Lives, 2010). Morbidity and mortality rates are rising faster in developing countries; inequalities in health access within particular country favor the growth of such rates (Human Development Report 2013, 2013). The chronic illnesses make a profound impact on patients and society, regarding complex and expensive treatment strategies, decreased quality of life and working disability. Most of them are treatable, but not curable, which additionally prolongs the enormous influence on the health funds (Jakovljevic and Milovanovic, 2015; EU Reflection on Chronic Disease, 2016). The objectives of this study were to estimate the impact of drug utilization related to NCDs on Serbian health funds, and compare it with the Gross National Income (GNI) per capita values for the observed period.

## Non-communicable diseases in Serbia

Although, the life expectancy at birth for both sexes was increased by 3 years over the period of 2000–2012, in 2012 the loss of healthy life expectancy due to morbidity and disability was 9 years lower than overall life expectancy at birth (Country Statistics Global Health Estimates by WHO UN Partners, 2015; Jakovljevic et al., 2015a). Significant increase in treating cardiovascular diseases (CVDs) contributed to such progress, although the adult risk factors as raised blood glucose, obesity, tobacco usage and raised blood pressure are slightly higher in Serbia then the European average (Possible Directions for Increasing Efficiency of Healthcare System in Serbia, 2015). Mortality range for CVDs reached 762–744.9 per 100,000 population for a 10 years period (2004–2013), 259.4–294.4 for malignant neoplasms, 34.4–39.1 for diabetes and 34.2–36.0 for chronic obstructive pulmonary disease (COPD; Health statistical yearbook of Republic of Serbia, 2013). NCDs in Serbia are estimated to account for 95% of all deaths, which is significantly higher compared to the 86% of the European region (EU Reflection on Chronic Disease, 2016). Mortality due to cardiovascular diseases reached 54% of all deaths, followed by cancers (23%), COPD (4%), and diabetes (3%) (Noncommunicable Diseases (NCD) Country Profiles, 2015).

## The data report methods—databases

Data obtained from the World Bank and OECD National Accounts files concerning Serbia (Serbia, 2016) and from the publications *Marketing and consumption of medicinal products for human use of Medicines and Medical Devices Agency of Serbia* (ALIMS) were observed for each year of a ten-year period (2004–2013) (Marketing and consumption of medicinal products for human use (2004–2013), 2016). The quantum of Gross National Income per capita, obtained by Atlas method and originally expressed in currency US$, was observed and converted into euros using yearly average exchange rates for each year. GNI per capita presents the gross national income, converted to U.S. dollars using the World Bank Atlas method, divided by the mid-year population. GNI is the sum of value added by all resident producers plus any product taxes that were not included in the valuation of output plus net receipts of primary income from abroad.

Medicine consumption for pharmacotherapy of the most common NCDs and financial expenses were calculated based on the World Health Organization (WHO) ATC classification, for the following groups of drugs: A08-Antiobesity preparations, excl. diet products, A10-Drugs used in diabetes, B01-Antithrombotic agents, C-Cardiovascular system, L-Antineoplastic and immunomodulating agents and R03-Drugs for obstructive airway diseases. Expenses were expressed in euros (EUR), consumption of medicines in Defined Daily Doses per 1000 inhabitants per day (DDD/1000 inh/day) and ratios as a percentage of a total quantum (%).

## Drug utilization regarding the GNI

**Figure 1:**
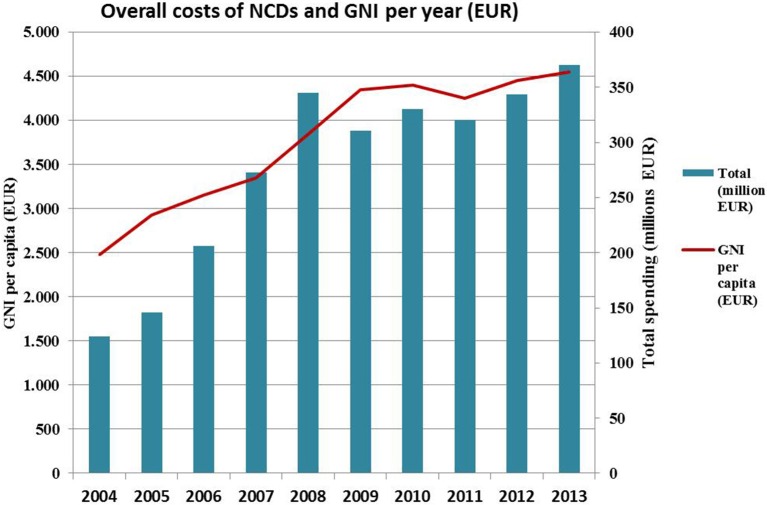
**Overall costs for pharmacotherapy of the most common NCDs compared with GNI for the selected time period**. It can be observed that the expenses for medicines followed the GNI trend line, expressing an even more rapid growth of the financial assets in years 2007 and 2008.

Values of GNI per capita in Serbia recorded a rapid growth, starting from 2004 to 2010, when a slight regression occurred with a modest re-growing tendency in the period from 2011 to 2013. The expenses for indicated medicines followed the GNI trend line, expressing an even more rapid growth of the financial assets, especially in 2007 and 2008 (Figure 1; Jakovljevic et al., 2015b).

In every particular year within the observed period, the greatest funds were dedicated to the pharmacotherapy of cardiovascular diseases. In the year 2008, it reached the highest share of 25.5% regarding total medicine expenses. In the terms of drug utilization in DDDs/1000 inh/day, the highest consumption was in 2013 with the share of 44.38% of all prescribed medicines.

The funds for antineoplastic and immunomodulating agents almost doubled their share within the observation period, reaching the highest values in 2013, both in the financial and utilization terms.

The expenses for drugs used in diabetes nearly tripled their value in 2013 in comparison to 2004, whereby that was not the case with the relative ratio regarding entire medicine funds, which has remained more or less constant. The consumption and its ratio followed the above-mentioned path of expenses (Jakovljevic and Souliotis, 2016).

The drugs for obstructive airway diseases are at the fourth place among pharmacotherapy resources, with the constant growth in both terms, reaching the peak of the expenses and consumption in 2011, in the nominal values as well as in ratio terms.

The prescription of antithrombotic agents shows an increasing tendency, together with increasing costs of treatment, while decrease of consumption and consequently costs cutting concerning antiobesity preparations, excl. diet products, was noticed.

## Pharmacotherapy data compared to the cost of illness

Approximately 10% of Serbian GDP was spent on the total health care, thereof the share of government expenditure amounted 62%. Total pharmaceutical market in Serbia, with included prescription and over-the-counter medicines, is estimated at €645.6 million, with per capita expenditure estimated at €89.37. National health insurance funds for medicines amounted €45.32 per capita in 2012, and reached 18% of total budget. Considering the fact that NCDs are the most common cause of death, current morbidity and mortality rates have to be reduced in order to maintain sustainability of health care funds (Godman and Gustafsson, 2014).

As a part of NCDs, cardiovascular diseases impose a significant burden on the society, not only in terms of morbidity and mortality, but also as a significant economic impact. Amongst CVDs, stroke remains the main cause of death, followed by cardiomyopathy and ischemic heart disease; however, those diseases show no changes in the crude death rate in 2012 compared to 2000. Hypertensive heart disease shows growing tendency in the same time period (Noncommunicable Diseases (NCD) Country Profiles, 2015). In order to estimate the economic burden of cardiovascular diseases, a study conducted in Serbia referring year 2009, evaluated their direct and indirect costs from the point of view of the society. Total costs reached 514.3 million euros, the greatest part of which was influenced by medications (29.94%; Lakić et al., 2014). According to the ALIMS data, for the cardiovascular system disorders medicines only, it was necessary to refund between 80 and 180 million Euros per each observed year (2004–2013; Table 1). If the expenses of the antithrombotic agents are added to the cardiovascular medicines, we can conclude that the cardiovascular diseases are a highly costly group of diseases with a heavy burden on the society. It is even more if we consider the fact that the total cost of illness amounted to approximately 1.8% of the Serbian GDP (Lakić et al., 2014).

**Table 1:** **Expenses (EUR) and consumption of the specific medicine groups for the selected years and ratios regarding their total spending and consumption (%)**.

**Year**	**Expenses (EUR)**	**% of total spending on medicines**	Consumption (^*^DDD/1000 inh/day)	**% of total consumption**
**C—CARDIOVASCULAR SYSTEM**				
2004	78.716.560,68	23,20	331,02	30,54
2005	83.534.063,75	21,94	334,63	30,57
2006	122.294.456,31	23,94	387,81	38,26
2007	158.399.085,91	23,04	400,06	36,89
2008	203.799.903,76	25,50	481,70	39,51
2009	161.297.625,34	21,74	414,67	35,21
2010	162.352.661,21	22,89	384,73	39,82
2011	163.667.924,49	22,66	474,31	42,94
2012	176.571.683,75	23,80	526,98	42,44
2013	182.656.566,45	22,99	568,28	44,38
**L—ANTINEOPLASTIC AND IMMUNOMODULATING AGENTS**				
2004	19.166.161,32	5,65	1,52	0,14
2005	23.112.621,92	6,07	1,49	0,14
2006	30.880.478,61	6,05	2,12	0,21
2007	49.424.984,02	7,19	2,32	0,21
2008	60.977.545,27	7,63	2,32	0,19
2009	68.839.374,51	9,28	2,71	0,23
2010	77.515.115,27	10,93	2,38	0,25
2011	68.873.960,86	9,54	2,19	0,20
2012	75.968.428,70	10,24	3,32	0,27
2013	89.944.696,11	11,32	3,61	0,28
**A10—DRUGS USED IN DIABETES**				
2004	13.673.374,33	4,03	15,88	1,46
2005	21.607.559,81	5,68	27,51	2,51
2006	23.647.892,69	4,63	41,82	4,13
2007	28.566.352,17	4,15	41,18	3,80
2008	35.355.225,48	4,42	59,68	4,90
2009	30.357.067,89	4,09	49,31	4,19
2010	34.022.142,79	4,80	45,73	4,73
2011	36.770.569,28	5,09	45,19	4,09
2012	37.668.755,11	5,08	74,50	6,00
2013	42.739.770,26	5,38	79,97	6,25
**R03—DRUGS FOR OBSTRUCTIVE AIRWAY DISEASES**				
2004	8.758.308,28	2,58	18,40	1,70
2005	9.751.546,83	2,56	17,47	1,60
2006	16.558.281,51	3,24	26,87	2,65
2007	16.745.056,96	2,44	23,09	2,13
2008	19.043.385,18	2,38	25,53	2,09
2009	24.353.872,40	3,28	18,40	1,56
2010	26.190.593,19	3,69	23,17	2,40
2011	26.784.686,01	3,71	23,22	2,10
2012	22.604.678,59	3,05	15,06	1,21
2013	25.425.617,85	3,20	18,55	1,45
**B01—ANTITHROMBOTIC AGENTS**				
2004	2.994.811,70	0,88	11,00	1,01
2005	7.020.869,89	1,84	19,80	1,81
2006	11.245.999,68	2,20	29,08	2,87
2007	18.433.025,18	2,68	55,44	5,11
2008	24.131.961,20	3,02	54,32	4,46
2009	24.750.838,47	3,34	69,16	5,87
2010	29.334.215,64	4,14	58,82	6,09
2011	23.823.928,51	3,30	67,94	6,15
2012	30.130.846,05	4,06	73,34	5,91
2013	29.289.470,21	3,69	89,68	7,00
**A08—ANTIOBESITY PREPARATIONS, EXCL. DIET PRODUCTS**				
2004	711.589,90	0,21	0,10	0,01
2005	1.033.497,60	0,27	0,17	0,02
2006	1.313.206,95	0,26	0,22	0,02
2007	1.320.202,45	0,19	0,23	0,02
2008	1.413.316,40	0,18	0,25	0,02
2009	1.295.245,35	0,17	0,30	0,03
2010	524.148,20	0,07	0,10	0,01
2011	474.353,52	0,07	0,09	0,01
2012	402.346,66	0,05	0,08	0,01
2013	402.979,73	0,05	0,08	0,01
	**Total medicines expenses(EUR)**		Total ^*^DDD/inh/day	
2004	339.279.303,77		1.083,97	
2005	380.716.701,39		1.094,61	
2006	510.833.609,54		1.013,70	
2007	687.588.174,80		1.084,34	
2008	799.082.221,00		1.219,07	
2009	741.981.960,19		1.177,72	
2010	709.317.344,16		966,26	
2011	722.207.154,57		1.104,57	
2012	742.013.975,72		1.241,66	
2013	794.560.044,61		1.280,47	

* *DDD/1000 inh/day, Defined Daily Doses per 1000 inhabitants per day*.

Ischemic heart disease is the leading cause of mortality and morbidity, expressed in terms of disability-adjusted life years (DALY). In Europe, ischemic heart disease accounted for 24.8% of all the DALYs caused by other diseases, while in Serbia it accounts for only 1% less (23.8%). In highly developed countries, the mortality rate of ischemic heart disease has been decreasing, due to better socio-economic conditions, increased health protection service quality and healthy life styles promotion (Jakovljevic et al., 2016). At the same time, mortality is high in the east-European countries. In 2009, standard mortality rate for Serbia amounted to 113.8 compared to the European Union average of 87.2 per 100,000 inhabitants (Mickovski et al., 2011). Serbian age standardized death rate regarding CVDs in 2014 of 400.8 per 100,000 males, was amongst highest in the whole European region; e.g., in United Kingdom it amounted 140.6, Denmark 134.6 and France 111.8 per 100,000 males (Global status report on noncommunicable diseases 2014, 2014).

Calculated from the point of view of the society, the peculiarity in the Serbian cost of illness calculations would be the fact that only one fourth of all the expenses were made up of indirect costs. That differs from the developed countries data concerning the treatment of CVDs in which indirect costs would reach almost half of the total costs (Chan et al., 1996; Liu et al., 2002).

Worldwide, cancer represents an important public health and economic issue, as the global cancer incidence in 2012 was 14.1 million with 8.2 million death cases (Worldwide cancer statistics, 2014). In 2014 the age standardized death rate from cancers in Serbia reached 218.1, while in UK, Denmark and France it amounted 153.9, 179.9, and 179.8 per 100,000 males, respectively (Global status report on noncommunicable diseases 2014, 2014). Cancer treatment costs impose considerable expenses on the national health system budgets, reaching in average 9.1% of the Gross Domestic Product Expenditure on Health (GDPHE) for developed European countries, up to 17.1% in USA in 2010 (Pritchard et al., 2016). On average, 10% of total health care costs in developed countries are also spent on treating cancer (Bosanquet and Sikora, 2004).

Differences in costs occur not only due to different types of cancer, but also as a consequence of the stage of the disease (Dagovic et al., 2014). Based on the research conducted in the central Serbian region the same authors concluded, that in the total costs of cancer care the medication share reached 5%, while malignant breast neoplasm turned out to be the most expensive overall treatment. For the patients with the advanced stage of cancer, the greatest impact on total medical treatment costs was observed concerning medicines expenditures (Kovacevic et al., 2015a). Significant costs are obtained for colorectal cancer pharmacotherapy, depending upon the stage of cancer. In Serbian settings, it was necessary to pay from 4200 to 20,600 Euros per patient with metastatic disease (Kovacevic et al., 2015b). The data obtained from the ALIMS show a significant fivefold increase in the expenses and more than twofold increase of consumption of antineoplastic and immunomodulating agents in 2013 compared to 2004 (Jakovljevic et al., 2014a).

The cost of diabetes mellitus, with its high morbidity and mortality rates, imposes significant burden on the national healthcare system of Serbia. The Institute of Public Health of Serbia mortality estimates were 39.1 per 100,000 population, but morbidity data show the presence of almost half a million people with this illness (Health statistical yearbook of Republic of Serbia, 2013). Compared to some European countries, e.g., Greece, the death rate equals only 6.6 per 100,000 males (Global status report on noncommunicable diseases 2014, 2014). The theoretical cost of handling this disease would even reach 6% of the total Serbian health care expenditure or 11% of the annual Health insurance fund budget (Jakovljevic, 2014). In real life, these costs would be considerably lower, since not all the population is covered by health insurance. Data concerning developed countries, such as Switzerland, show that 2.2% of the total health care expenditure is spent on the specific diabetes care (Schmitt-Koopmann et al., 2004), while in the United States 10–20% of the health budgets are spent on diabetes mellitus treatment (American Diabetes Association, 2008). Based on the research conducted by Biorac et al. (2009), medicine share in overall costs of illness for the year 2007 reached 38% in Serbia. An increasing trend in insulin and its analogs as well as oral antidiabetics consumption was observed. The ALIMS showed a fourfold increase in drug consumption for diabetes treatment and a threefold increase in expenses in 2013 in comparison to 2004. Antidiabetic medication share in the overall consumption showed a rapid growth in the observed period, but that was not the case with the costs, whose share in total expenses was only 5.38% in 2013 compared with 4.03% in 2004 (Table 1).

As a severe disabling and irreversible illness, COPD is standing among top five most expensive chronic disorders due to its high healthcare budget impact. Its estimated prevalence in developed countries makes 4–6% among adult males as well as 1–3% among females, with smoking as a dominant risk factor. The COPD patients often have different severe comorbidities that rise costs of treatment and make distinction from the basic illness difficult. The greatest contributing factors in overall treatment costs are periodic exacerbations of the disease, which are getting higher with the COPD severity degree (Andersson et al., 2002). The major cost drivers in the developed countries would be physician consultations and surgery, while in the middle-income eastern countries, pharmaceuticals and oxygen costs are dominant. In Serbia, the incidence of COPD mortality is almost equal to the one of the diabetes (Health statistical yearbook of Republic of Serbia, 2013). COPD related pharmaceutical prescription reached the peak of the value turnover in 2011, with almost 27 million Euros, while the consumption of such medicines remained constant, with lesser pop outs, in the entire observational period (Table 1). The similar situation was observed in the entire western Balkan market, when value of medicines for the treatment of the respiratory diseases rose to 46.5 million Euros in 2012. COPD comorbidities significantly contributed to the greater costs of medical care, e.g. costs of community acquired pneumonia (CAP) clinical treatment accounted for an average 717 Euros in a 1 month, while COPD and CAP together accounted for 970 Euros during the same time period (Cupurdija, 2015). According to the results from a recent published study, concerning comparison of two different markets, Serbian and Belgian, drug acquisition costs (in- and outpatient consumption) of COPD treatment reached up to 54% in Serbian and 46% in Belgian sample. The greatest difference actually occurred in the relative number of hospital bed days and hospital admissions due to exacerbation of the disease, per person, which were significantly smaller in Belgian population (Jakovljevic et al., 2013).

Obesity is present in the Serbian population in 24.8% of the adult population. This ratio is increasing; some forecasts predict that in 2020, 44% of men and 31% of women will be obese (Nutrition, Physical Activity and Obesity, 2013). Consumption and expenses of medicines concerning this disease are not following the predicted path, since their significant decrease occurred in 2008, making only 0.01% of total drugs consumption as well as 0.05% of the overall expenses (Table 1). We strongly support other anti-obesity measures, such as promoting healthy life style, which would make a significantly higher contribution in the management of this problem, rather than pharmacotherapy (Jakovljevic and Ogura, 2016).

## Conclusion

Almost 80% of all deaths due to chronic diseases like CVDs, cancer, COPD and diabetes occur in the low- and middle-income countries. The rapid aging of the Serbian population (Jakovljevic and Laaser, 2015), with higher demands for the healthcare services, along with the difficulties in transitional national health insurance fund functioning, have resulted in the huge impact on the national budget as well as household expenditures (Ogura and Jakovljevic, 2014).

This data report shows that the total pharmacotherapy costs of treating CVDs in Serbia followed the GNI trend line, with noticeably constant growth of the financial assets. Expenses of medicines reached highest values in 2008 and 2013, even overgrowing the GNI rising trends.

Together with measures of prevention, chronic disease morbidity and mortality can be significantly reduced if pharmacotherapy is accessible and affordable. There have to be more initiatives and real-life activities in order to alleviate one's access to the treatment, as well as to improve governmental interventions aiming to facilitate the affordability of the drugs used to treat chronic diseases (Jakovljevic et al., 2015c).

One of the efficient measures could involve expanding the National Health Insurance Fund Drug List, both with brands and generic equivalents (Jakovljevic et al., 2014b). In addition, a certain part of health funds must be dedicated to substantial promotion of healthy life styles and minimizing verified risk factors for the occurrence of NCDs.

Possible limitations of the study would likely be the underestimated drugs utilization and pharmacotherapy costs for the particular diseases. In treating most NCDs, many different medications are used, not only those within specified ATC groups, but for other concomitant diseases, which could elevate calculated costs. Calculations did not comprehend the out-of-pocket expenses for different Over the Counter medicines due to no published data regarding their consumption. Drug prices data within the ALIMS publications database are calculated using regulatory approved drug prices, not the real values ensued from the special terms of sale.

## Author contributions

All the authors listed have made substantial contribution to the conception, design, analysis and interpretation of data for the work, and approved it for publication. AK, VD developed research questions, designed the study and prepared the manuscript. NR, ZS searched the literature, analyzed and interpreted the data for the work. VD, ZS critically reviewed the manuscript.

### Conflict of interest statement

The authors declare that the research was conducted in the absence of any commercial or financial relationships that could be construed as a potential conflict of interest.
